# Editorial: Why Livestock Genomics for Developing Countries Offers Opportunities for Success

**DOI:** 10.3389/fgene.2020.00626

**Published:** 2020-06-26

**Authors:** Farai C. Muchadeyi, Eveline M. Ibeagha-Awemu, Ardeshir N. Javaremi, Gustavo A. Gutierrez Reynoso, Joram M. Mwacharo, Max F. Rothschild, Johann Sölkner

**Affiliations:** ^1^Agricultural Research Council-Biotechnology Platform, Pretoria, South Africa; ^2^Agriculture and Agri-Food Canada (AAFC), Sherbrooke, QC, Canada; ^3^Department of Animal Science, University of Tehran, Tehran, Iran; ^4^Programa de Mejoramiento Animal, Universidad Nacional Agraria La Molina, Lima, Peru; ^5^International Center for Agricultural Research in the Dry Areas (ICARDA), Addis Ababa, Ethiopia; ^6^Department of Animal Science, Iowa State University, Ames, IA, United States; ^7^Department of Sustainable Agricultural Systems, Division of Livestock Sciences, University of Natural Resources and Life Sciences, Vienna, Austria

**Keywords:** indigenous livestock, genetic adaptation, genomics, production systems, developing countries

In the developing world, rural farmers rely on local breeds to play crucial roles aimed at ameliorating the effects of adverse environments and resources shortages in sustaining their livelihoods. The local breeds appear to be adapted to numerous unfavorable environmental stressors that include worsening droughts characterized by extreme temperatures and debilitating disease challenges, the epitome of low input production systems. Breeding and genetics research programs are striving to develop robust animals that are adapted to local conditions and can produce at optimal and sustainable levels under constrained environments. Elucidating the intertwined relationship between production environments and the genetics of animals, with the aim of establishing selection priorities and developing suitable improvement strategies, is critical. Previously, livestock improvement programs have failed to realize expected gains due to the lack of performance data, pedigree records and funding, and worsened by such factors as uncontrolled livestock breeding practices on communal pastures. Advances in livestock genomics have facilitated the generation of “big data” in genetics through the advent of whole genome/transcriptome sequencing, genome assemblies and genome-wide SNP genotyping. Regardless of the room for genetic gains in local breeds and the anticipated higher impact of genomics assisted breeding and selection, developing countries still lag behind in the uptake of genomic technologies. This Research Topic addresses the need for livestock genomics for developing countries through review articles, original research articles and considerations of future opportunities.

The Research Topic yielded 23 articles that are either review (five papers) or original research articles (18 papers) covering major livestock species kept in developing countries including cattle (seven papers), sheep (five papers), goats (three papers), and chickens (three papers). The manuscripts cover a broad range of genomic applications such as genomic selection/assisted breeding, genome-wide association analysis, diversity studies with a particular emphasis on adaptive genetic variation and signatures of selection analysis, and some elements of functional genomics using RNA sequencing and differential gene expression profiling. Whilst a broad range of genomic applications are covered, there is a bias toward genomic diversity studies, indicating the limited utility of other genomic applications due to inherent limitations to data collection and funding that characterize most developing countries, and are highlighted in some of the review articles.

The reviews provide an overview of the current and potential applications of genomics in developing countries, the opportunities that can be used from other supporting technologies such as reproductive technologies and the challenges and possible solutions of applying genomics in a developing country context. According to Mrode et al., genotypic data can provide solutions for parent verification, breed composition determination and genetic evaluation for smallholder farmers. The review by Mrode et al., also highlights the major problem of small reference populations, which could be overcome by across regional genomic prediction programs that pull together data from multiple countries. The review by Ducrocq et al. explores challenges facing developing countries, including limited capacity to genotype, poor data management, multiple breeding goals emanating from exposure to unfavorable conditions such as heat and diseases, requirement of special attention on fitness traits and limited expertise to drive genomics programs. Marshall et al. present case studies from Africa on the application of livestock genomics which included the identification and development of unique breeds in the region. This review also looks at the role of genomic studies on African livestock to understand the genetics of particular diseases and in the potential of technologies such as gene editing in disease management. The review by Van Marle-Kőster and Visser highlight the benefits of a dual system of a highly developed commercial sector using the most recent technologies vs. a small holder and communal sector in South Africa, and how resources can be harnessed to advance both sectors. This review also highlights the importance of national animal recording schemes and government funding to ensure progress in driving the application of genomics across the two sectors. In line with this, Ibeagha-Awemu et al. discuss the importance of leveraging available resources and stakeholder involvement for coordinated improvement of livestock production in Africa. The review further highlights in-depth approaches that can enable the application of genomic technologies for rapid improvement of livestock traits of economic importance in the era of genomic breeding.

The first set of original research papers present case studies of genomic selection and genome-wide association analysis. Hosseini-Vardanjani et al. evaluate the gain in accuracy of genomic evaluations using multi-breed reference populations and demonstrates the utility of incorporating prior knowledge of principal components in genomic prediction as well as the potential of a multi-breed reference population to contribute to enhanced prediction accuracies. In the absence of conventional genetic evaluations and selection, Mujibi et al. and Cheruiyot et al. use genomic data to understand breed composition and associate it to production performance. Genome-wide association studies are often challenging because of the need of very large number of experimental units with good phenotypes. A genome-wide association study (GWAS) by Xu et al. highlights the potential for different genetic mechanisms for litter size among sheep breeds. Nazari-Ghadikolaei et al. identify candidate genes for coat color and mohair traits in the Iranian Markhoz goats through a GWAS. Bhuiyan et al. use imputed sequence level SNP data in a GWAS to identify variants in genic and exon regions significantly associated to carcass traits in Korean Hanwoo cattle.

The second set of original research articles describes the common application of genomics in smallholder livestock systems of developing countries such as analysis of the level of admixture and investigation of signatures of selection in cattle (Chagunda et al.; Alshawi et al.); sheep (Ahbara et al.; Al-Atiyat et al.; Edea et al.); goats (Onzima et al.; Cui et al.) and in native chickens (Elbeltagy et al.; Walugembe et al.; Lawal et al.). Finally, Pierce et al. investigate copy number variations (CNVs), which have recently gained prominence, as a genomic tool, to ascertain genetic diversity and population structure in South African cattle.

Only one study focuses on functional genomics, using RNA-Seq and differential gene expression studies to investigate genetic and molecular mechanisms underlying traits of importance in sheep (Ma et al.). This probably reflects the complexities of setting up transcriptome experiments in largely uncontrolled smallholder farming systems of developing countries.

Overall, the topic demonstrates the utility of genomics in diverse application across species and geographical regions of the developing countries and the opportunities that exist in the future.

## Author Contributions

FM and JS initiated the Research Topic and invited MR, EI-A, AJ, GG, and JM as topic co-editors. FM drafted the Research Topic editorial. All authors participated in the editorial process of this Research Topic, revised, and approved the final draft of the editorial.

## Conflict of Interest

The authors declare that the research was conducted in the absence of any commercial or financial relationships that could be construed as a potential conflict of interest.
